# Experiences and perceptions of physical healthcare among adult autistic patients: A scoping review

**DOI:** 10.1016/j.ijnsa.2025.100366

**Published:** 2025-06-10

**Authors:** Åsa Hedlund, Anna Andersson, Magnus Lindberg, Malin Jordal

**Affiliations:** Department of Caring Sciences, University of Gävle, Kungsbäcksvägen 47, Gävle 801 76, Sweden

**Keywords:** Autism, Inequality, Somatic healthcare

## Abstract

**Background and purpose:**

The number of people diagnosed with autism is increasing globally, and autism is associated with poorer health outcomes. However, there is a lack of knowledge regarding how physical healthcare is experienced and perceived by adult autistic patients. The purpose of this scoping review is to provide an overview of the research on adult autistic patients’ experiences and perceptions of physical health care.

**Results:**

Sixteen articles from four databases were included. They originated from the US, Europe, and Australia. Most of the articles were published between 2022 and 2024 and employed qualitative, quantitative, or mixed-methods approaches. Adult autistic patients’ experiences and perceptions of physical healthcare concerned various aspects, including the healthcare organization, rooms and spaces, healthcare staff, and physical examinations and treatments. The patients need opportunities to engage in written communication with healthcare staff, as well as waiting room environments that are low-sensory in nature.

**Conclusions:**

Overall, the findings indicated that the healthcare needs of adult autistic patients were not being met, particularly concerning communication and sensory impressions. Additionally, there is a need for further research on inpatient hospital care as well as physical examinations and treatments.

**What is already known about the topic**

• Autistic patients experience sensory overload in healthcare settings, but this knowledge is often based on data from psychiatric care and children.
• Healthcare professionals describe caring for autistic patients as challenging, because they lack knowledge about autism.

Alt-text: Unlabelled box

**What this paper adds**

• The main challenges in physical healthcare for the adult autistic patients involved communication, sensory perceptions, and the need for predictability.
• Physical examinations/treatments involving elements of surprise were experienced as highly uncomfortable by the adult autistic patients.

Alt-text: Unlabelled box

## 1 Introduction

Among adult patients in physical healthcare (i.e., healthcare services that address physical health needs, including prevention, diagnosis and treatment), autistic patients have been poorly researched. The existing research on autism has primarily focused on psychiatric care, even though physical care is a much more common form of care for people in a society. Autism is common worldwide, and the number of diagnosed people is increasing (Centers for Disease Control and Prevention, 2024). In 2023, it was estimated that between 2 and 3 % of all children in the US have the diagnosis (Centers for Disease Control and Prevention, 2024). The prevalence is lower among adults, probably due to undetected cases, and not because there are fewer autistic adults than children (O'Nions et al., 2023). Autism is always present during childhood, and it is a lifelong condition (American Psychiatric Association, 2024). Autistic people socially interact and communicate in ways that differ from most people. Moreover, they exhibit restricted and repetitive behavior and interests that may manifest as a need for predictability and strict routines in daily life (American Psychiatric Association, 2024). Being hypersensitive to sensory inputs such as sounds or visual patterns is also a common trait (American Psychiatric Association, 2024). It is also common for autistic people to have a bottom-up thinking process, meaning that they build a whole from knowledge of details, rather than through previous experiences. For this reason, autistic individuals rarely 'assume' things, but must understand things anew each time using the information available at the moment (Sanborne, 2024). However, autism exists along a spectrum, meaning that it encompasses a wide range of symptoms, abilities, and challenges, varying in severity and presentation across individuals (American Psychiatric Association, 2024).

It has been shown that people in the autistic community prefer to be called “autistics” rather than individuals with autism, as autism should not be perceived as a pathological condition but rather as a part of their identity (Walker, 2024; Taboas et al., 2023). For this reason, in the present study we use the term “adult autistic patients” instead of “adult patients with autism.”

Autistic people tend to have poorer physical health and shorter lifespans than non-autistic people, which can be attributed to unhealthy lifestyle habits and less frequent utilization of healthcare services (World Health Organization, 2023). Research has shown that autistic patients (including psychiatric care, adolescents and children) may experience sensory overload in healthcare settings, especially regarding auditory and visual stimuli. For example, one Swedish study focusing on healthcare settings as a whole revealed that autistic adults, compared to non-autistic adults, reported greater difficulties tolerating sounds like phones ringing, TV sounds and alarms (Strömberg et al., 2022). They also felt misunderstood by healthcare professionals and found it difficult to communicate with the professionals due to their lack of knowledge about autism. For example, staff could ask open-ended questions instead of more specific ones or fail to understand that the experience of something like pain may be different for autistic people (Strömberg et al., 2022). Healthcare professionals have, in turn, described caring for autistic patients as challenging, because they feel they lack knowledge about autism as well as the resources needed to provide the best care (Morris et al., 2019). This may form the basis for unequal care between autistic and non-autistic patients.

Interventions to improve healthcare for autistic patients do exist, but the focus is often on autistic children (Harris et al., 2024). There are a few newly established primary care centers in the US designed specifically for autistic patients, and studies have shown that these patients report a higher level of satisfaction with their care compared to autistic patients in regular primary care (Hand et al., 2020). In addition to the staff's high level of knowledge about autism, these centers offer features such as shorter waiting times, longer appointment durations, and flexibility in communication (e.g., patients can choose to communicate in writing instead of verbally). However, this only pertains to primary care, and physical healthcare is much broader than that.

To understand and meet the needs of adult autistic patients in physical healthcare, we first need to gain knowledge of their experiences and perceptions of such care. The aim of the present scoping review was therefore to provide an overview of the research on adult autistic patients’ experiences and perceptions of physical healthcare. The research questions were:

1. What are the characteristics of the studies that have examined the experiences and perceptions of autistic patients regarding physical healthcare?
2. What are adult autistic patients’ experiences and perceptions of physical healthcare?

## 2 Methods

The present scoping review was guided by the methodology of Arksey and O'Malley (2005), which suggests the following steps: (a) identify the research questions; (b) identify relevant studies; (c) study selection; (d) charting, synthesizing, sorting the data; and (e) collating, summarizing and reporting the results. Additionally, the PRISMA 2020 checklist was followed to ensure clear reporting of methodology and results in the present review (Page et al., 2021).

### 2.1 Search strategy

Articles were searched for in March 2024 in Medline via EBSCO, CINAHL, Scopus and Web of Science, with the help of an experienced university librarian. It was a requirement that the articles be published in scientific journals from the 2014 onwards and be peer-reviewed, criteria that were used as a filter in the databases during the database searches. The searches focused on autistic adults (population) and experiences and perceptions of physical healthcare (outcome). To include studies with adult autistic patients, the initial search was for autism (e.g., "autism" and "autism spectrum disorder") followed by adults (e.g., "women" and "men"). To search for experiences of physical healthcare, search terms for experiences and perceptions (e.g., experiences, perceptions, perspective and preference) and physical healthcare (e.g., "medical care" and "hospital") were used. See Appendix for a detailed description of the search strategy.

### 2.2 Study selection

Of the 4396 records identified in the databases Medline via EBSCO, CINAHL, Scopus and Web of Science, duplicates (*n* = 1367) were identified and removed by the librarian using Rayyan, which is a web-based tool designed to assist in the screening and selection of studies for systematic reviews. This left 3029 articles. The screening process was inspired by Mateen et al. (2013); titles alone were screened first, because this has been shown not to affect the final outcome, as compared to screening both title and abstract. The inclusion and exclusion criteria are presented in Table 1.

**Table 1:** An overview of the inclusion- and exclusion criteria.

Inclusion criteria	Exclusion criteria
• Qualitative, quantitative or mixed methods studies • Adults (≥18 years old) with autism • *Experiences* (i.e., something the autistic adult has personally undergone or lived through) and/or *perceptions*, (i.e., how the autistic adult interprets or views something) of physical healthcare. • Physical healthcare, i.e., healthcare services that address physical health needs, including prevention, diagnosis and treatment.	• Other perspectives than those of adult autistic patients, such as healthcare professionals’ or relatives’ perspectives. • Psychiatric/mental healthcare • Dental care • Childbirth and maternity care • Children (<18 years) • Reviews and other non-empirical articles • Other language than English • Studies based on specialized autism-focused care facilities^1^ • Experiments/intervention studies • Perception of proposed recommendations for improving care • Finances/insurance and transportation routes to healthcare • Psychometric studies

1 To avoid selection bias and ensure that the results can be generalized to standard healthcare.

**Fig. 1:**
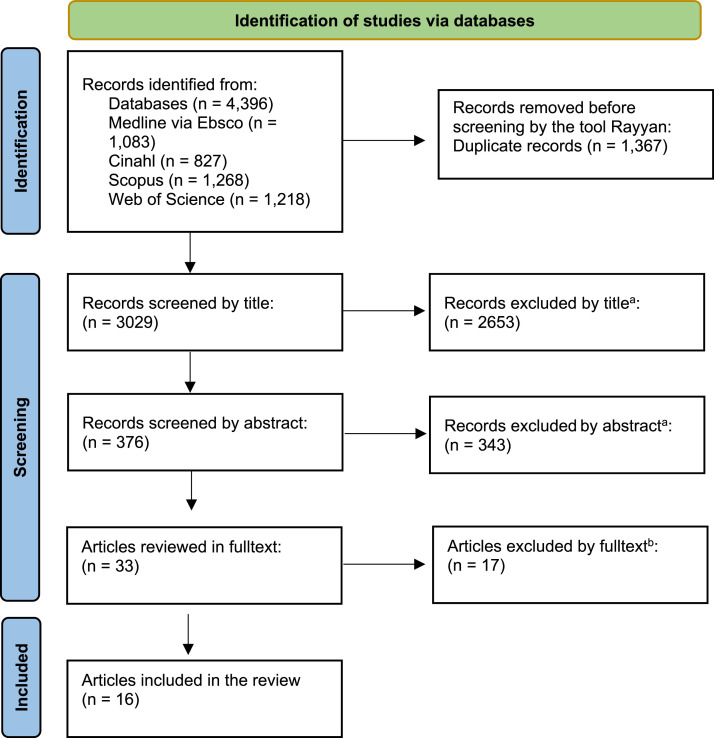
PRISMA flowchart depicting the selection process. a. Main reasons for exclusion: Wrong publication type (e.g., reviews), wrong population (e.g., parents) and wrong healthcare setting (e.g., psychiatry). b. Reasons for exclusion: The participants were too young, impossible to distinguish relevant participants from irrelevant ones, it was unclear what applied to somatic care, data was taken from patient records instead of from the autistic individuals themselves; foreign languages; focused on a specific autism-centered facility.

ÅH reviewed the titles to determine whether they seemed relevant for inclusion, considering the inclusion and exclusion criteria for the present review. After this step, 376 articles remained. The authors (ÅH, AA, ML, MJ) reviewed the abstracts of the remaining studies and discussed the relevance of the studies for the review. This resulted in 33 possible articles. Then, ÅH and AA read these 33 articles in full and discussed them with ML and MJ (who also read the articles in full text) when they were unsure about their relevance for the review. Finally, 16 articles were included in the study. A flowchart depicting the selection process is shown in Fig. 1.

### 2.3 Data analysis

We undertook an inductive analysis inspired by Peters et al.’s (2020) and Pollock et al.’s (2023) recommendations concerning how to perform data analysis for systematic reviews. A detailed description of our procedure is presented below.

*Analysis of Research Question 1:* First, we decided what aspects of the studies we wanted to present in the results (e.g., type of physical healthcare, outcome) and created a data matrix into which these selected parts of the results could be entered. Then, AA, MJ and ML distributed the articles between them and extracted the demographic data (e.g., type of physical healthcare, country) from the articles, entering the data into the matrix. Patterns in the demographic data were then looked for to be able to present the data in a narrative and organized manner in the results.

*Analysis of Research Question 2:* Two independent reviewers (ÅH and AA) identified data in the articles’ respective result sections regarding adult autistic patients’ experiences and perceptions of physical healthcare. After this, they met and compared the data they had identified, and in cases of disagreement, they discussed the data until reaching a consensus on which data should be included in the present review and why. Then, these data were extracted from the articles, entered into a data matrix and read thoroughly several times by ÅH to identify units of analysis, i.e., segments of text that were considered to have a single message. We decided not to analyze experiences and perceptions separately, because they are difficult to distinguish from each other. Each unit of analysis was then labelled with a code, answering the question “What is this data unit about?” in relation to physical healthcare. The codes were created to achieve a low level of interpretation and abstraction. For example, sensitivity to sounds and sensitivity to light were labelled “sensory impressions.” The codes were then organized based on similarities and differences, which enabled the creation of categories.

ÅH led the analysis process, but throughout it, all authors discussed with each other several times until consensus was reached regarding interpretation of data and naming of categories.

## 3 Results

### 3.1 Study characteristics

**Fig. 2:**
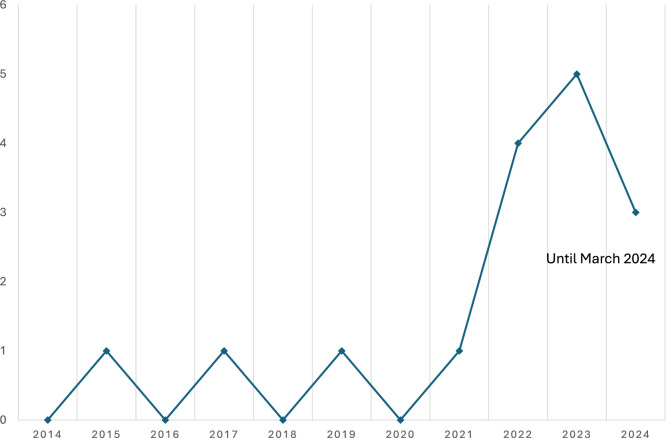
Overview of number of publications a year from 2014 until March 2024.

The present review was based on 16 studies published between 2015 and 2024. There is a trend toward a growing interest in the field, as only a few studies were conducted before 2022. See Fig. 2 for a more detailed view of the studies’ respective publication years.

**Fig. 3:**
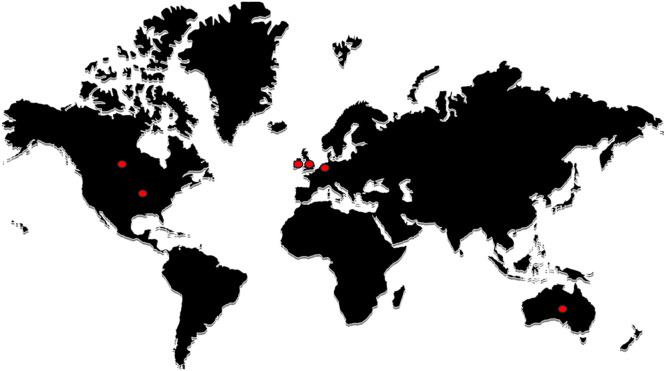
An overview of the geographical origin of the included studies.

Most studies (*n* = 9) did not specify the type of healthcare involved, but they seemed to involve various types of outpatient clinics. Among the studies specifying type of healthcare, primary care was the most common (*n* = 5). The remaining studies focused on optometry (*n* = 1) and gender identity healthcare (*n* = 1). Most studies were from the UK (*n* = 5) and the US (*n* = 4). The rest of the studies were from Canada (*n* = 1), The Netherlands (*n* = 1) and collaborations between Belgium and Australia (*n* = 2), the UK and The Netherlands (*n* = 1), the UK, the US and Ireland (*n* = 1), the UK and the US (*n* = 1), and Belgium and The Netherlands (*n* = 1). See Fig. 3 for an overview of the distribution of the studies around the world.

Men, women and non-binary patients were represented in the included studies. Not everyone had a formal autism diagnosis; some declared themselves autistic or exceeded the questionnaire cutoff for being considered autistic. Seven of the included studies had a qualitative approach (Bruce et al., 2023; Calleja et al., 2022; Mazurek et al., 2023; McLean et al., 2024; Nicolaidis et al., 2015; Taylor et al., 2023; Parmar et al., 2022), six had a quantitative approach (Arnold et al., 2024; Brice et al., 2021; Featherstone et al., 2022; Shaw et al., 2024; Duker et al., 2019; Vogan et al., 2017), and three studies used both approaches (Warreman et al., 2024; Maljaars et al., 2023; Mason et al., 2022). See Table 2 for details regarding study characteristics.

**Table 2:** Article characteristics.

Author, title, year	Country	Design and analysis	Type of somatic healthcare	Study sample	Main findings regarding autistic adults’ experiences and perceptions of somatic healthcare
Arnold, S. R., Bruce, G., Weise, J., Mills, C. J., Trollor, J. N., & Coxon, K. Barriers to healthcare for Australian autistic adults. 2024	Australia	Cross-sectional Quantitative Independent samples *t*-tests Correlations Linear regression	No specific focus on somatic care	A total of 263 autistic and 70 non-autistic australian adults aged 25 years or over who completed the ALSAA self-report survey at the second timepoint. The autistic group was comprised of individuals with either a formal or self-diagnosis, who were included due to the underdiagnosis of autism historically	• Waiting rooms are uncomfortable • It is difficult to communicate with the healthcare staff • Healthcare staff misunderstand you • Not taken seriously by healthcare staff • Not allowed to be involved in discussions about one's own care • Difficult to understand the healthcare system • Difficult to book an appointment • Healthcare providers do not offer enough support • Anxiety and shame prevent visits • Sensitivity to sensory stimuli complicates care • difficulty understanding how to translate medical information into concrete steps that one can take to improve one’s health • Problems filling out paperwork • Difficult to identify and describe symptoms, which affects communication with healthcare staff • More autistic individuals than non-autistic individuals experienced barriers to healthcare, and they also experienced more barriers than the non-autistic individuals.
Brice S, Rodgers J, Ingham B, Mason D, Wilson C, Freeston M, Le Couteur A, Parr JR. The importance and availability of adjustments to improve access for autistic adults who need mental and physical healthcare: findings from UK surveys. 2021	United Kingdom	Cross-sectional Quantitative Independent *t*-tests Chi-square tests Exploratory factor analysis Polychoric correlations Descriptive statistics McNemar’s comparisons	No specific focus on somatic care	UK-based autistic adults registered with the Adult Autism Spectrum Cohort-UK. Recruitment was staged over a 12-month period. 537 autistic adults completed a survey about mental health services (51 % response rate), 407 completed the physical health survey (49 % response rate). 221 participants completed both surveys.	• It's important not to have too many sensory stimuli in the premises • It must be easy to find the right place • Want a low noise level in the premises • Want there to be only a few people in the premises • It is important that healthcare staff have knowledge about autism • There are often no accommodations for autism • It is important to be able to communicate with healthcare digitally • Short wait times are important • Most of the time, it is a familiar place you are going to • Want the opportunity to ask questions at the end of the visit • Adjusted length of the appointment is important • Want to recognize the healthcare staff. • Healthcare staff sometimes listen to the patient's needs • Important to receive autism-friendly accommodations in care • Want the opportunity to provide information about oneself in advance • Want the opportunity to stim (repetitive movements used by autistic individuals to self-soothe or regulate sensory input) • Want access to distractions (e.g., a tablet) during the waiting time
Bruce H, Munday K, Kapp SK. Exploring the Experiences of Autistic Transgender and Non-Binary Adults in Seeking Gender Identity Health Care. 2023	United Kingdom (Researchers) Participants Australia, Canada, England, Germany, Norway, and The Netherlands	Explorative Qualitative Reflexive thematic analysis	Gender identity health care	17 adults with a formal or self-diagnosed autism, at least 18 years old, and having first-hand experiences of accessing or trying to access gender identity health care as a transgender or non-binary person.	• Noisy waiting rooms are stressful • Dim lighting in the healthcare environment is pleasant • The healthcare staff should show more interest in person-centered care for autism • Healthcare staff need more knowledge about autism • Healthcare staff infantilize you • Healthcare staff need to explain things better • Uses own strategies to get the desired care • Healthcare staff ask intrusive questions • Difficult to find information • Lack of alternative communication methods besides phone contact • Inaccessible healthcare provider • Long wait times negatively affect well-being
Calleja, S., Kingsley, J., Amirul Islam, F. M., & McDonald, R. Barriers to Accessing Healthcare: Perspectives from Autistic Adults and Carers. 2021	Australia	Phenomenological Qualitative Thematic analysis	No specific focus on somatic care (but probably primary health care)	Data were collected from a sample of autistic adults (*n* = 9) Autistic adults and primary caregivers were included in this study if they (1) were aged 18 years or older, (2) they lived in Melbourne Australia and (3) have a diagnosis of autism.	• The physical environment of the premises is important for the care experience • Healthcare staff need to explain more • Healthcare staff's low knowledge about autism negatively impacts care • Healthcare staff ask unclear questions • Not heard by the healthcare staff • Sensitivity to sensory stimuli complicates care
Featherstone C, Sharpe RA, Axford N, Asthana, S, Ball S, Husk K. Barriers to healthcare and their relationship to well-being and social support for autistic adults during COVID-19. 2022	United Kingdom	Longitudinal Quantitative Rpeated measures ANOVA Linear regression modelling Chi-square tests	No specific focus on somatic care	One hundred twenty-eight participants completed the initial survey. 89.1 % of the sample reported having a clinical diagnosis of autism. Forty-two participants (39 % of those re-contacted) completed the follow-up questionnaire.	• Waiting rooms are uncomfortable • Difficult to communicate with healthcare staff • Misunderstood and not taken seriously by healthcare staff • Not allowed to be involved in discussions about one’s own care • Does not understand the healthcare system • Healthcare providers do not offer enough support according to one's needs • Anxiety and shame hinder seeking care • Sensitivity to sensory stimuli complicates care • Difficult to identify and describe symptoms to healthcare staff • Difficulty understanding how to translate medical information into concrete steps that one can take to improve health
Maljaars J, Gijbel E, Evers K, Spain D, Rumball F, Happé F, Noens I. Impact of the COVID-19 Pandemic on daily life: Diverse experiences for autistic adults. 2023	Belgium, The Netherlands, The United Kingdom	Exploratory longitudinal online survey Quantitative and qualitative Independent samples *t*-tests and Chi Squares Thematic analysis	No specific focus on somatic care	424 participants. 317 Dutch-speaking adults (220 females, 96 males, 1 ‘other’) aged 18–74 years (*M* = 41.6), and 107 English-speaking adults (77 females, 20 males, 10 ‘other’) aged 19–73 years (*M* = 41.3). The Dutch-speaking participants were living in Belgium (*n* = 265), The Netherlands (*n* = 50) and the United Kingdom (*n* = 2), and the English-speaking participants were all living in the United Kingdom (*n* = 107). The 424 participants were divided into two different groups. The autism group (*n* = 196) comprised 161 individuals with a formal diagnosis of autism and 35 individuals self-identifying as having autism. The non-autism group consisted of 228 individuals without an autism diagnosis or self-identification. In both groups, most participants were female (70 %). The age ranged from 18 to 74 years old, and age was equally distributed between both groups.	• Feel that the healthcare system is under pressure and thus wait too long to contact them • Prefer digital meetings over in-person ones • Both autistic and non-autistic individuals experienced challenges within healthcare, but these challenges were perceived as worse (more negative) among the autistic individuals.
Mason, D., Taylor, H., Ingham, B., Finch, T., Wilson, C., Scarlett, C Urbanowicz, A., Nicolaidis, C., Lennox, N., Moss, S., Buckley, C., Cooper, SA., Osborne, M., Garland, D., Rymarker, D. and Parr, JR. Views about primary care health checks for autistic adults: UK survey findings. 2022	United Kingdom (England and Wales)	Cross-sectional Quantitative Qualitative Chi-square tests Content analysis	Primary health care	A questionnaire was sent to autistic adults with physical health conditions in England and Wales. A total of 458 people (441 autistic adults and 17 proxy responders) completed the questionnaire. Recruitment was through the Adult Autism Spectrum Cohort-UK (ASC-UK), ^25^ an ongoing, longitudinal study of the lived experiences of UK autistic adults; any UK-based autistic person aged ≥16 years is able to participate in ASC-UK.	• Having few sensory stimuli in the waiting room is positive • Healthcare staff need to have knowledge about autism • Condescending healthcare staff complicate care • Healthcare staff's lack of knowledge about autism complicates care • Healthcare staff do not listen to you • Want written information from the healthcare staff • Want to communicate digitally • There is lack of adjustments for autism in healthcare • Want to have healthcare staff that you recognize • Want detailed information about the upcoming visit
Mazurek, M. O., Sadikova, E., Cheak-Zamora, N., Hardin, A., Sohl, K., & Malow, B. A. Health Care Needs, Experiences, and Perspectives of Autistic Adults. 2023		Phenomenological Qualitative Phenomenological analysis	No specific focus on somatic care	Twenty autistic adults (aged 18–35 years, 65 % male) completed surveys and individual semi-structured interviews.	• Noisy, chaotic environments are problematic • Rooms with fewer sensory stimuli promote well-being • Healthcare staff need to explain more and easier • Healthcare staff are sometimes skilled at balancing involvement of relatives and person-centered care • Healthcare staff needs to have a more person-centered approach • Difficult to communicate verbally with healthcare staff • Lack of knowledge about autism among healthcare staff results in negative consequences for care • Healthcare staff are good at making small talk in a relaxing way • Feels rushed during visits, which negatively affects communication • Healthcare staff show interest, which is good for the care experience • Want to avoid phone calls • Waiting for a long time in waiting rooms is stressful • Wants to be able to book appointment online • Want the same healthcare staff every time • Sometimes get appointments quickly and other times they need to wait a long time • Difficult to manage anxiety and pain during visits • Sensitivity to sensory stimuli complicates care • Brusque examinations lead to refusal to agree to them • Stimming (e.g., squeeze something that feels satisfying) helps during exams • Humor from healthcare staff during examinations is important • Uncomfortable to have reflexes examined (when the leg 'kicks up')
McLean KJ, Haas M, Koenig J, Horvath M, Vigil M, Werner NE. Bishop L. "I'm dealing with a health care system that doesn't get it": Barriers and facilitators to inclusive healthcare for autistic adults. 2024	United States	Descriptive Qualitative Thematic analysis	No specific focus on somatic care	23 autistic adults (age 18+; (2) had an administrative, professional, or suspected diagnosis of autism spectrum disorder (ASD); and (3) able to communicate in English).	• Noise and clutter in the premises are problematic • Sitting in large open waiting areas hinder decision-making • Difficult to navigate in the premises • Healthcare staff do not take the time to listen • Healthcare staff lack knowledge about autism • Healthcare staff misunderstand you • Want to avoid phone calls and be able to book appointments online • Want to know what to expect • Difficult to understand when healthcare staff speak quickly and interrupt • Fear of healthcare visits due to concerns about what the healthcare staff will think of you • Sensitivity to sensory stimuli complicates care • Must have a well-thought-out approach to get the desired care • Anxiety increases when exposed to too many sensory stimuli
Nicolaidis, C., Raymaker, D. M., Ashkenazy, E., McDonald, K. E., Dern, S., Baggs, A. E., Kapp, S. K., Weiner, M., & Boisclair, W. C. “Respect the way I need to communicate with you”: Healthcare experiences of adults on the autism spectrum. 2015	US	Qualitative Thematic analysis	No specific focus on somatic care	Our final sample of 39 autistic adults, Age (in years) Mean 35 (range 19–64) Participants had to be US residents, at least 18 years of age, and communicate in written or spoken English or American Sign Language,. Additionally, the primary sample needed to report a formal diagnosis of autism, Asperger’s, pervasive developmental disorder not otherwise specified, or autism spectrum disorder.	• Receiving sensory stimuli in the healthcare environment is positive • Healthcare staff underestimate you intellectually • Healthcare staff cannot handle the need to communicate in atypical ways • Healthcare staff lack knowledge about autism • It is important that the staff involve relatives when needed, which they sometimes do • Openness to accommodations by the care provider facilitated interaction between you and the healthcare staff • Want continuity in care • The larger organization, not just the staff etc., affects the experience of care • It is difficult to understand the healthcare system • Has a slow processing speed in thoughts, which affects communication with healthcare staff • Sensitivity to sensory stimuli complicates care • Trying to hide one's autism to prevent healthcare staff from viewing you negatively • Difficult to interpret symptoms, which affects communication with healthcare staff
Parmar KR, Porter CS, Dickinson CM, Baimbridge P, Pelham J, Gowen E. Autism-friendly eyecare: Developing recommendations for service providers based on the experiences of autistic adults. 2022	United Kingdom	Explorative Qualitative Study 1: Four focus groups, each with four to six participants Thematic analysis Study 2: One-to-one interviews Qualitative content analysis using an inductive approach	Optometry services	Study 1: 18 autistic adults, aged 18–67. Mean age 47.1 years. Six were female. All had a formal diagnosis of an autism spectrum condition., were from the north of England and had previously visited an optometrist. Study 2: 24 autistic adults, aged 19–67. Mean age 43.3 years. 14 identified as male, nine as female and one as non-binary. All had a formal diagnosis of an autism spectrum condition, were from the north or southeast of England.	• Important to have calm and quiet environments without too intense lights • Healthcare staff need to speak slowly • Difficult when the healthcare staff ask many different or vague/open questions • Helpful when healthcare staff explain what will happen, and offer visual explanations • Different healthcare staff and switch rooms are stressful • Helpful to be able to book appointments and get reminders digitally • Uses writing beforehand as a strategy to keep the conversation brief • Uncomfortable when healthcare staff touches you • Examination equipment can give the feeling of being trapped • Examinations could be a nice experience to because of the equipment used, and the examination's uniform structure • Want to be able to control the pace of the examination
Shaw, S. C., Carravallah, L., Johnson, M., O'Sullivan, J., Chown, N., Neilson, S., & Doherty, M. Barriers to healthcare and a ‘triple empathy problem’ may lead to adverse outcomes for autistic adults: A qualitative study. 2023	UK USA Ireland	Explorative Qualitative Thematic analysis	Primary health care	This article reports data relating to autistic adult respondents. Both formal diagnosis and self-identity were acceptable. One thousand two hundred and forty-eight autistic adults responded to the surveys. The majority lived in the United Kingdom (*n* = 571), followed by the United States (*n* = 226) and Ireland (*n* = 206).	• Healthcare staff misunderstand you • Feels devalued by healthcare staff • Sympathetic and understanding healthcare staff reduce anxiety • Unexpectedly long wait times in the waiting room cause anxiety • Hides their autism to be taken seriously • Needs a well-thought-out communication strategy to get the desired care • Afraid of being seen as a bad person
Stein Duker LI, Sadie Kim HK, Pomponio A, Mosqueda L, Pfeiffer B. Examining Primary Care Health Encounters for Adults With Autism Spectrum Disorder. 2019	United States	Descriptive cross-sectional questionnaire. Descriptive statistics	Primary health care	34 adults with autism spectrum disorder whereof 28 males, having a score ≥65 on the Ritvo Autism Asperger Diagnostic Scale revised. 31 Caregivers	• Environment in waiting rooms can be stressful • Healthcare staff misunderstand you and don't take you seriously • Lack of support from healthcare • Adaptations work well when they are offered • Sensitivity to sensory impressions makes things more difficult
Taylor H, Ingham B, Mason D, Finch T, Wilson C, Scarlett C, Moss S, Buckley C, Urbanowicz A, Raymaker D, Seiboth C, Lees R, Garland D, Osbourne M, Lennox N, Cooper SA, Nicolaidis C, Parr JR. Co-design of an NHS primary care health check for autistic adults. 2023	United kingdom	Experienced based co-design Qualitative Reflexive thematic analysis using an inductive approach	Primary health care	31 autistic adults including 6 with a co-occurring intellectual disability, 8 adults with intellectual disability who were not autistic.	• Healthcare staff need to explain more • Healthcare staff need to involve relatives without losing person-centeredness • Healthcare staff need to gather information in a way that feels easy • Healthcare staff lack knowledge about autism • Healthcare staff ask too many questions • Prefer to communicate in writing rather than by phone • Easier if you have an appointment booked rather than having to take the initiative and book one yourself • Want to know what will happen • Difficult to understand and describe one's own symptoms to healthcare staff
Vogan V, Lake AK Tint A. Weiss JA, Lunsky Y. Tracking healthcare service use and the experiences of adults with autism spectrum disorder without intellectual disability: a longitudinal study of service rates, barriers and satisfaction. 2017	Canada	Longitudinal survey Quantitative Descriptive statistics Bivariate analyses	No specific focus on somatic care	Participants included 40 adults who self-reported a clinical diagnosis of Asperger Syndrome (AS) or ASD without ID.	• Not understanding the healthcare system is a barrier to seeking care • Fear that the healthcare staff will think negative things about you prevents seeking care • Negative experiences of healthcare prevent future care seeking
Warreman EB, Wietske A, Geurts HM, Vermejren RR, Nootebom LA. How do primary care providers and autistic adults want to improve their primary care? A Delphi study. 2024	The Netherlands	Delphi study Qualitative Quantitative Thematic analysis Descriptive statistics	No specific focus on somatic care	Interviews. For the semi-structured interviews, two groups were recruited: (1) people who receive care (*n* = 5) and (2) care providers (*n* = 6). Adults, with a minimum age of 18years old, with a self-reported autism spectrum disorder (ASD) diagnosis or with an autistic child were included in the first group. In the second group, care providers working with autistic patients in primary, secondary or tertiary care were eligible for inclusion. Delphi-study. The Delphi-panels also consisted of two groups: (1) autistic adults and (2) PCPs. We aimed to include approximately 20 participants in each group,	• If healthcare staff have negative prejudices about autism, lack knowledge of autism or are not interested in autism, that is barriers to good caregiving • Enough time for the visit is important • Important that different parts of the healthcare system can collaborate • Difficulties for the autistic individuals such as communication, changes, coping with emotions or being sensitive to sensory stimuli complicates care

### 3.2 How experiences and perceptions have been investigated

The quantitative studies used questionnaires to collect data on autistic adults’ experiences and perceptions of physical healthcare. The most frequently used questionnaire was Barriers to Healthcare Checklist (Arnold et al., 2024; Featherstone et al., 2022; Duker et al., 2019). Other questionnaires focused on the impact of Covid-19 on contact with health/support services (on a scale from 1-5) (Maljaars et al., 2023) and satisfaction with service (on a scale from 1-5) (Vogan et al., 2017). The items on one questionnaire served as a health check for autistic adults (Mason et al., 2022) and one survey explored barriers to healthcare (Shaw et al., 2024; Warreman et al., 2024).

The qualitative studies were based on interview questions focused on one phenomenon, such as experiences of an eye examination (Parmar et al., 2022), experiences of gender identity healthcare (Bruce et al., 2023), views on health checks (Taylor et al., 2023), perceptions concerning barriers to healthcare (Warreman et al., 2024), how and why autistic adults make decisions about interfacing with healthcare and what their experiences with healthcare settings are (McLean et al., 2024), experiences of healthcare access (Calleja et al., 2022), experiences and perceptions of receiving healthcare (Mazurek et al., 2023), and general healthcare experiences (Nicolaidis et al., 2015). See Table 2 for a more detailed overview.

### 3.3 Experiences and perceptions of physical healthcare

**Fig. 4:**
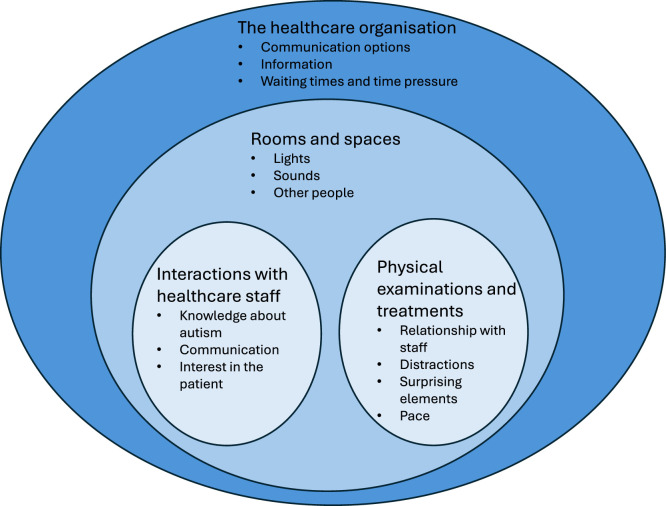
An overview of the categories and their main content.

This part of the results is presented using four categories; see Fig. 4 for an overview of the categories and their main content. The figure illustrates how the categories range from the macro-level to the micro-level within the healthcare system. The categories pertain to the overall healthcare organization, including the rooms and spaces where encounters with healthcare staff, examinations, and treatments take place. Below, the categories are described in more detail.

#### 3.3.1 The healthcare organization

The healthcare organization, i.e., the structural aspects within an organization that influence its function, were highlighted in 15 of the studies (Arnold et al., 2024; Brice et al., 2021; McLean et al., 2024; Mazurek et al., 2023; Bruce et al., 2023; Parmar et al., 2022; Featherstone et al., 2022; Nicolaidis et al., 2015; Mason et al., 2022; Duker et al., 2019; Warreman et al., 2024; Taylor et al., 2023; Vogan et al., 2017; Shaw et al., 2024; Maljaars et al., 2023). Patients experienced the healthcare system as difficult to understand, inaccessible, burdensome and inflexible in relation to autistic patients’ needs (Arnold et al., 2024; Taylor et al., 2023; Bruce et al., 2023;Featherstone et al., 2022; Brice et al., 2021; Duker et al., 2019; Vogan et al., 2017; Nicolaidis et al., 2015). Several patients mentioned the need for clear information about what to expect during the visit. They felt such information should be available in various formats, such as video and written text (Parmar et al., 2022; Mason et al., 2022). They also wanted more opportunities to communicate or to schedule healthcare appointments digitally (McLean et al., 2024; Mazurek et al., 2023; Bruce et al., 2023; Maljaars et al., 2023; Taylor et al., 2023; Mason et al., 2022; Parmar et al., 2022; Brice et al., 2021), mainly because they found it difficult to speak on the phone, as this required being able to verbally express oneself well and concisely. The patients described continuity as important (Warreman et al., 2024; Parmar et al., 2022; Mason et al., 2022; Brice et al., 2021; Nicolaidis et al., 2015), referring to visiting the same facilities and/or seeing the same staff over time. If continuity was lacking, they could experience such stress over the changes and unpredictability that they avoided seeking care even when they needed it. If they did experience continuity, however, they felt comfortable during the appointment (Mazurek et al., 2023).

Several patients described issues related to time (Warreman et al., 2024; Shaw et al., 2024; Mazurek et al., 2023; Bruce et al., 2023; Maljaars et al., 2023; Mason et al., 2022; Brice et al., 2021; Duker et al., 2019), i.e., long and unpredictable waiting times (for months at home or minutes/hours in the waiting room at the clinic – both of which were perceived as stressful) as well as time pressure during the actual appointment. The latter was described as negatively affecting the quality of care because there was not enough time to discuss things the patient felt were important to discuss. If patients sensed the staff were under time pressure, they could, in addition to feeling stressed themselves, feel they were a burden to the staff and therefore hesitate to seek care at a later stage (Maljaars et al., 2023).

#### 3.3.2 Rooms and spaces

Experiences and perceptions of physical rooms and spaces, such as waiting rooms and consultation rooms, were described in 11 of the included studies (Arnold et al., 2024; Brice et al., 2021; McLean et al., 2024; Mazurek et al., 2023; Bruce et al., 2023; Parmar et al., 2022; Featherstone et al., 2022; Nicolaidis et al., 2015; Mason et al., 2022; Calleja et al., 2022; Duker et al., 2019).

The emphasis was mainly on the importance of reducing the noise, light and number of people in the rooms. Loud and surprising noises (e.g., phones ringing) and strong and surprising lights (e.g., flashes, reflections) were perceived as uncomfortable and overwhelming (McLean et al., 2024; Bruce et al., 2023; Mazurek et al., 2023; Mason et al., 2022; Parmar et al., 2022; Brice et al., 2021; Nikolaidis et al., 2015). Waiting rooms full of people and other sensory stimuli could make it difficult for the autistic patient to know where to go, which was experienced as highly stress-inducing (McLean et al., 2024). A room that was perceived as stressful and overwhelming could complicate communication with the healthcare staff because the sensory impressions caused distractions and thus a lack of concentration (Bruce et al., 2023). As a result of this, patients expressed a wish to be in rooms where there were only a few people, dim lights and little noise. Some participants described how they had experienced these kinds of comfortable stress-reducing examination rooms, which they perceived as calming (Bruce et al., 2023).

#### 3.3.3 Interactions with healthcare staff

In 15 of the included studies (Arnold et al., 2024; Brice et al., 2021; McLean et al., 2024; Mazurek et al., 2023; Bruce et al., 2023; Parmar et al., 2022; ; Nicolaidis et al., 2015; Mason et al., 2022; Calleja et al., 2022; Duker et al., 2019; Warreman et al., 2024; Taylor et al., 2023; Vogan et al., 2017; Shaw et al., 2024), experiences and perceptions related to the healthcare staff were described.

Most of the studies reported how adult autistic patients experienced a lack of knowledge about autism among healthcare staff (Arnold et al., 2024; McLean et al., 2024; Shaw et al., 2024; Warreman et al., 2024; Bruce et al., 2023; Taylor et al., 2023; Mazurek et al., 2023; Calleja et al., 2022;Featherstone et al., 2022 Mason et al., 2022; Parmar et al., 2022; Duker et al., 2019; Nikolaidis et al., 2015). This negatively affected a person-centered approach and participation in care. Lack of knowledge could manifest as underestimating the person’s intelligence (e.g., treating them like a child, talking only to relatives, being patronizing), not explaining what the patient can expect during the visit or having little flexibility in ways of communicating (e.g., not accepting written communication or asking too vague or too many questions). Fear of being negatively judged by healthcare personnel was described (McLean et al., 2024; Shaw et al., 2024; Featherstone et al., 2022), and this had prevented some autistic patients from seeking care. However, it was not only healthcare personnel who were perceived as uncomprehending. One patient in Shaw et al.’s study (2024) described how the receptionist at the healthcare setting had an unfriendly and uncomprehending attitude, which negatively affected the patient's experience of the healthcare visit.

Several adult autistic patients described difficulties in communicating verbally with the staff (Arnold et al., 2024; Bruce et al., 2023; Mazurek et al., 2023; Taylor et al., 2023; Calleja et al., 2022; Parmar et al., 2022), both because they found it hard to find the right words and because they struggled to process what the staff were saying or asking. Patients experienced that staff spoke too rapidly, extensively and in a complicated manner. Several participants reported having altered their behavior and having tried to camouflage their autism to reduce the risk of staff thinking negatively about them (Shaw et al., 2024; Nicolaidis et al., 2015). However, this camouflaging could not hide all the difficulties.

Several patients described having had difficulty identifying, interpreting and describing symptoms, which made it hard to communicate symptoms to the healthcare staff (Taylor et al., 2023; Nicolaidis et al., 2015). However, patients who prepared a visual/written description of their needs before the appointment found that this could facilitate communication with healthcare staff (Duker et al., 2019). The staff, however, were not always interested in looking at patients’ visual/written descriptions (Nikolaidis et al., 2015). Some patients reported requiring better explanations of what will happen during the visit and why (Taylor et al., 2023; Mazurek et al., 2023; Calleja et al., 2022; Parmar et al., 2022). Not knowing what to expect made patients feel unsafe, and not understanding the reasons for actions led to a lack of motivation to comply with any suggestions given. Simple explanations, sometimes with the help of images, could improve patients’ ability to understand (Mazurek et al., 2023; Parmar et al., 2022).

Patients described positive experiences of healthcare staff who seemed interested, showed respect (e.g., not only talking to the patient’s relatives), were attentive and met their communicative needs (e.g., offered visual explanations) (Shaw et al., 2024; Mazurek et al., 2023; Parmar et al., 2022).

#### 3.3.4 Physical examinations and treatments

Experiences and perceptions of physical examinations and treatments were described in two studies (Parmar et al., 2022; Mazurek et al., 2023). These examinations/treatments could be perceived as both pleasant (e.g., certain materials could be perceived as satisfying) and unpleasant (Parmar et al., 2022). What contributed to making the examination or treatment pleasant was having a good relationship with the staff (e.g., staff using humor or already being familiar with the patient) (Mazurek et al., 2023; Parmar et al., 2022), being allowed to distract oneself and calm down by touching a texture that gave tactile satisfaction (Mazurek et al., 2023). Having had previous positive experiences with the examination or treatment was also helpful (Parmar et al., 2022). What could contribute to making the examination or treatment unpleasant or anxiety-inducing were sensory stimuli such as unwanted touch and strong odors from the healthcare staff (Parmar et al., 2022). Surprising elements in the examination, such as checking knee reflexes or eye pressure (“sudden air puff” toward the eyeball), were also described as uncomfortable (Mazurek et al., 2023; Parmar et al., 2022). Being involved in and being able to influence the pace of the examination or treatment were important to feeling safe and comfortable (Mazurek et al., 2023; Parmar et al., 2022).

## 4 Discussion

The present scoping review included 16 articles from three continents: North America, Europe and Australia. Most articles were published between 2022 and 2024 and mainly focused on primary care or other kinds of outpatient care. The results on adult autistic patients’ experiences and perceptions of physical healthcare showed that they faced several challenges in relation to such healthcare, ranging from organizational issues to physical examinations and treatments. The main challenges involved communication, sensory perceptions, and the need for predictability. It was important to have alternative ways to communicate with healthcare services and staff beyond verbal communication, as well as to be seen as a capable adult worthy of being taken seriously despite being autistic. A calm healthcare environment regarding visual impressions, sounds, and lighting was important, as was avoiding overcrowded waiting rooms. It was also important to have the same healthcare staff over time to build a trusting relationship, as well as to receive sufficient information (and in the right way) about what to expect during the visit.

The results show that there are challenges at both the macro-level (the organization) and micro-level (the interaction between patient and healthcare staff). However, at both levels, they largely revolved around communication. The adult autistic patients expressed a desire for the organization to provide alternative methods of communication in addition to verbal ones (McLean et al., 2024; Mazurek et al., 2023; Bruce et al., 2023; Maljaars et al., 2023; Taylor et al., 2023; Mason et al., 2022; Parmar et al., 2022; Brice et al., 2021), and for healthcare staff to communicate “better” with the autistic patients (e.g., treating them like intelligent adults and explaining things in more detail) (Arnold et al., 2024; McLean et al., 2024; Shaw et al., 2024; Warreman et al., 2024; Bruce et al., 2023; Taylor et al., 2023; Mazurek et al., 2023; Calleja et al., 2022; Featherstone et al., 2022; Mason et al., 2022; Parmar et al., 2022; Duker et al., 2019; Nikolaidis et al., 2015). Therefore, it can be concluded that, with regard to adult autistic patients, healthcare faces a communication challenge on multiple levels. Previous research among autistic adults has also shown that they prefer written communication, phone calls being the least favorable form of interaction (Howard and Sedgewick, 2021). Written communication was considered better because it allows more time to process what the other person is saying and to think about how to respond. Furthermore, written communication made it possible to choose a setting without overwhelming sensory stimuli in the environment, which could otherwise be stressful. Additionally, autistic patients saved energy by not having to think about masking—using the "right" facial expressions and tone of voice—which often does not come automatically to autistic individuals (Howard and Sedgewick, 2021). To reduce psychological stress and facilitate interactions with healthcare, it may therefore be highly beneficial for healthcare services to offer written communication options for autistic patients (or for all patients, as some may be undiagnosed autistics). For example, patients should be allowed to schedule appointments in writing and engage in a written dialogue with healthcare professionals about their health issues. Such digital solutions in healthcare are developing in several countries (OECD, 2023), and findings from the present literature review emphasize the importance of this for autistic patients.

According to the present findings, the sensory sensitivity associated with autism makes environments with crowds, noise, clutter and bright lights particularly challenging (Arnold et al., 2024; Brice et al., 2021; McLean et al., 2024; Mazurek et al., 2023; Bruce et al., 2023; Parmar et al., 2022; Featherstone et al., 2022; Nicolaidis et al., 2015; Mason et al., 2022; Calleja et al., 2022; Duker et al., 2019). Being particularly sensitive to sensory input is common among autistic people. Sensitivity to certain sensory inputs can occur in everyone, but it is often more intense and leads to more severe consequences among autistic people. This means that, for example, various sounds, lights, and visual patterns can create such intense discomfort that the autistic person feels the need to escape the situation to avoid severe anxiety, stress, and meltdowns (i.e., "mental breakdown") (Apex aba therapy, 2024). Therefore, for adult autistic patients, it is of utmost importance to reduce sensory inputs during healthcare visits. One way to achieve this is by offering virtual appointments, which have been shown to work well for adult autistic patients (Harris et al., 2022); this type of visit involves fewer sensory inputs because patients do not have to be in healthcare facilities.

However, not all types of healthcare visits can be conducted virtually, as a physical appointment is sometimes important. In those cases, it is essential to provide a low-sensory environment for adult autistic patients. One way to achieve this is by offering quiet spaces in healthcare settings (Greenwood et al., 2024). However, despite extensive research on the importance of the physical care environment in relation to well-being and person-centered care (Elf et al., 2024), autism has rarely been the focus. Black et al. (2022) explored how the physical environment in general, not specifically within healthcare, should be designed for autistic individuals. Their findings included aspects that may also be applicable to the built environment of healthcare settings, such as avoiding bright lights, implementing various methods to dampen loud noises in rooms, avoiding patterns on surfaces (e.g., striped walls), and providing the ability to regulate room temperature (to accommodate hypersensitivity or hyposensitivity to cold or heat).

Both the present literature review (Arnold et al., 2024; McLean et al., 2024; Shaw et al., 2024; Warreman et al., 2024; Bruce et al., 2023; Taylor et al., 2023; Mazurek et al., 2023; Calleja et al., 2022; Featherstone et al., 2022; Mason et al., 2022; Parmar et al., 2022; Duker et al., 2019; Nikolaidis et al., 2015) and previous research (Greenwood et al., 2024) have shown that healthcare professionals are perceived to lack knowledge about autism, something autistic patients see as a problem. For example, they may be treated as though they are less intelligent, or their needs may not be taken seriously. This is confirmed by findings from a review by Morris et al. (2019), where healthcare professionals reported a lack of knowledge about autism, including fundamental aspects such as diagnostic criteria, symptoms, and manifestations, as well as how to best adapt healthcare services to this patient group. Overall, this underscores the importance of increasing knowledge about autism among healthcare professionals. This could be achieved through university courses or internal training programs. For example, the present review (Taylor et al., 2023; Nikolaidis et al., 2015) as well as previous research (Strömberg et al., 2022) has indicated that autistic individuals express symptoms differently from the majority population. This is important for healthcare professionals to be aware of, and there is therefore a need for both more research and more training in this area to better identify the care needs of adult autistic patients in physical healthcare settings.

### 4.1 Future research

Most studies in the present review focused on primary care or other outpatient care. Therefore, there is a knowledge gap specifically regarding adult autistic patients’ experiences and perceptions of physical inpatient hospital care. Hospital care differs significantly from primary care. First, patients need to spend a longer time in the healthcare environment and have all their needs met there. Often, the patient is more seriously ill than in primary care and therefore has even greater care needs. Non-autistic people experience hospital stays to be highly challenging. For example, patients in Grondahl et al.’s (2013) study showed that hospital stays entail feelings of uncertainty, for example regarding routines and understanding what constitutes appropriate behavior in a hospital setting. It is reasonable to assume that autistic patients experience this as an even greater challenge, given their need for predictability and adapted communication styles. The results of the present review showed that, for adult autistic patients, sensitivity to sensory inputs such as sound, light, and smell was a stressor in the healthcare environment (Arnold et al., 2024; Brice et al., 2021; McLean et al., 2024; Mazurek et al., 2023; Bruce et al., 2023; Parmar et al., 2022; Featherstone et al., 2022; Nicolaidis et al., 2015; Mason et al., 2022; Calleja et al., 2022; Duker et al., 2019). Research is lacking on how sensory sensitivity manifests and is managed during an inpatient hospital stay. This is therefore an important area for further research. Following up on health and well-being after discharge from the hospital is also important. Does it take longer for autistic people, compared to non-autistic people, to recover after a hospital stay? Furthermore, what emerged in the present review that has not been clearly highlighted in previous research in this area is adult autistic patients’ experiences of physical examinations and treatments. Only two studies addressed this, and to a limited extent. Because physical healthcare largely involves various types of physical examinations and treatments, more research is needed on how these can be optimized for this patient group. However, it would seem that a high degree of predictability and distraction opportunities are important.

### 4.2 Limitations

The present study has some limitations. Because healthcare systems differ in various regions of the world, for example regarding accessibility, resources, and the organization of healthcare, the results may not be directly applicable in all countries. Another limitation is that the search was restricted to four databases. Although these databases contain a large number of articles in the healthcare field, an expanded search, including a manual search, may have resulted in a few more relevant articles. Excluding articles written in languages other than English also entails the risk of excluding relevant articles. Similarly, the exclusion of studies conducted in autism-specific healthcare settings is an important limitation of this review. While this decision was made to maintain a focus on mainstream service provision, it may have led to the omission of valuable insights into some practices and experiences, thus affecting the breadth and balance of the review’s findings. Last, it may be difficult to generalize the findings to adult autistic patients with an intellectual disability. Around one-third-of autistic people also have intellectual disability (Shenouda et al., 2023), and it is known that the proportion of autistic people with intellectual disabilities is lower in research contexts owing the complexity of giving informed consent and participating in data collection (McKenzie et al., 2023). Furthermore, it is important to note that the inclusion of self-identified autistic individuals entails a certain risk that non-autistic individuals are included in the study's results.

## 5 Conclusions

The present scoping review compiled knowledge on adult autistic patients' experiences and perceptions of physical healthcare. It makes an important contribution to educating healthcare personnel by helping them understand adult autistic patients’ needs in relation to physical healthcare, and it offers guidance on improving healthcare for adult autistic patients. The findings highlight the importance of reducing sensory stimuli in the healthcare environment, making visits as predictable as possible, offering alternative methods of communication other than verbal ones, and making it possible to see the same staff over time. However, the present review also reveals several knowledge gaps, particularly concerning inpatient hospital care and physical examinations and treatments. We suggest that more research be conducted in these areas.

## Funding

This research did not receive any specific grant from funding agencies in the public, commercial, or not-for-profit sectors.

## CRediT authorship contribution statement

**Åsa Hedlund:** Writing – original draft, Visualization, Project administration, Methodology, Formal analysis, Conceptualization. **Anna Andersson:** Writing – review & editing, Validation, Methodology, Formal analysis, Conceptualization. **Magnus Lindberg:** Writing – review & editing, Validation, Methodology, Conceptualization. **Malin Jordal:** Writing – review & editing, Validation, Methodology, Conceptualization.

## Declaration of competing interest

The authors declare that they have no known competing financial interests or personal relationships that could have appeared to influence the work reported in this paper.

## Appendix Supplementary materials

Image, application 1
