# Supplementary material for: Experiences and perceptions of physical healthcare among adult autistic patients: A scoping review
Source: Int J Nurs Stud Adv. 2025 Jun 10;9:100366. doi: 10.1016/j.ijnsa.2025.100366 (PMC12205838; doi:10.1016/j.ijnsa.2025.100366)
Supplement: Supplementary file 1 [file mmc1.docx]

| Database: Cinahl 2024-03-27 | | Items found |
| --- | --- | --- |
| **Autistic adults** | | |
| **1** | MH Autism Spectrum Disorder OR MH Autistic Disorder | 30,218 |
| **2** | TI (autism* OR autistic* OR "autism spectrum disorder") OR AB (autism* OR autistic* OR "autism spectrum disorder") OR SU (autism* OR autistic* OR "autism spectrum disorder") | 38,161 |
| **3** | S1 OR S2 | 38,161 |
| **4** | MH Infant OR MH Child OR MH Adolescent OR MH Minors | 626,575 |
| **5** | TI (Infant* OR infancy OR Newborn* OR Baby* OR Babies OR Child* OR Schoolchild* OR School age* OR Kid OR kids OR Toddler* OR Adoles* OR Teen* OR Boy OR boys OR boyfriend OR boyhood OR Girl* OR Minors OR minor) OR AB (Infant* OR infancy OR Newborn* OR Baby* OR Babies OR Child* OR Schoolchild* OR School age* OR Kid OR kids OR Toddler* OR Adoles* OR Teen* OR Boy OR boys OR boyfriend OR boyhood OR Girl* OR Minors OR minor) OR SU (Infant* OR infancy OR Newborn* OR Baby* OR Babies OR Child* OR Schoolchild* OR School age* OR Kid OR kids OR Toddler* OR Adoles* OR Teen* OR Boy OR boys OR boyfriend OR boyhood OR Girl* OR Minors OR minor) | 1,462,874 |
| **6** | S4 OR S5 | 1,462,874 |
| **7** | MH Adult | 1,305,465 |
| **8** | TI (adult* OR “middle aged” OR elderly OR women OR men OR woman OR man) OR AB (adult* OR “middle aged” OR elderly OR women OR men OR woman OR man) OR SU (adult* OR “middle aged” OR elderly OR women OR men OR woman OR man) | 2,065,463 |
| **9** | S7 OR S8 | 2,065,463 |
| **10** | S6 NOT S9 | 864,652 |
| **11** | S3 NOT S10 | 16,578 |
| **Experience of health care** | | |
| **12** | MH “attitude to health” OR MH “health communication” OR MH “health knowledge, attitudes, practice” OR MH “patient preference” OR MH “patient satisfaction” OR MH “health services accessibility” | 217,704 |
| **13** | TI (perspective* OR experience* OR attitude* OR need* OR emotion* OR affect OR communication OR access* OR comprehension OR perception* OR opinion* OR view* OR satisfaction OR preference OR barrier* OR obstacle* OR facilitator* OR expectation*) OR AB (perspective* OR experience* OR attitude* OR need* OR emotion* OR affect OR communication OR access* OR comprehension OR perception* OR opinion* OR view* OR satisfaction OR preference OR barrier* OR obstacle* OR facilitator* OR expectation*) OR SU (perspective* OR experience* OR attitude* OR need* OR emotion* OR affect OR communication OR access* OR comprehension OR perception* OR opinion* OR view* OR satisfaction OR preference OR barrier* OR obstacle* OR facilitator* OR expectation*) | 2,342,598 |
| **14** | TI (healthcare OR “health care” OR health-care OR “medical care” OR hospital* OR “health service*” OR “medical service*” OR “primary care” OR inpatient* OR patient* OR “acute setting” OR “acute care” OR “care setting” OR ward* OR “emergency department*” OR “emergency service*” OR “health facilit*” OR “health care facilit*”) OR AB (healthcare OR “health care” OR health-care OR “medical care” OR hospital* OR “health service*” OR “medical service*” OR “primary care” OR inpatient* OR patient* OR “acute setting” OR “acute care” OR “care setting” OR ward* OR “emergency department*” OR “emergency service*” OR “health facilit*” OR “health care facilit*”) OR SU (healthcare OR “health care” OR health-care OR “medical care” OR hospital* OR “health service*” OR “medical service*” OR “primary care” OR inpatient* OR patient* OR “acute setting” OR “acute care” OR “care setting” OR ward* OR “emergency department*” OR “emergency service*” OR “health facilit*” OR “health care facilit*”) | 3,328,472 |
| **15** | S13 N5 S14 | 560,101 |
| **16** | S12 OR S15 | 596,362 |
| **Combined sets** | | |
| **17** | S11 AND S16 | 1,137 |
| **Additional filters** | | |
| **18** | Academic Journals; Peer Reviewed; Publication Date (2014–) | 827 |

| Database: Web of Science Web of Science Core Collection (1986-present) 2024-03-25 | | Items found |
| --- | --- | --- |
| **Autistic adults** | | |
| **1** | TS=(autism* OR autistic* OR "autism spectrum disorder") | 108,285 |
| **2** | TS=(infant* OR infancy OR newborn* OR baby* OR babies OR child* OR schoolchild* OR school AND age* OR kid OR kids OR toddler* OR adoles* OR teen* OR boy OR boys OR boyfriend OR boyhood OR girl* OR minors OR minor) | 3,435,370 |
| **3** | TS=(adult* OR "middle aged" OR elderly OR women OR men OR woman OR man) | 4,480,066 |
| **4** | #1 NOT #2 | 37,898 |
| **5** | #1 NOT #4 | 70,387 |
| **Experience of health care** | | |
| **6** | TS=(perspective* OR experience* OR attitude* OR need* OR emotion* OR affect OR communication OR access* OR comprehension OR perception* OR opinion* OR view* OR satisfaction OR preference OR barrier* OR obstacle* OR facilitator* OR expectation*) | 15,297,296 |
| **7** | TS=(healthcare OR "health care" OR health-care OR "medical care" OR hospital* OR "health service*" OR "medical service*" OR "primary care" OR inpatient* OR patient* OR "acute setting" OR "acute care" OR "care setting" OR ward* OR "emergency department*" OR "emergency service*" OR "health facilit*" OR "health care facilit*") | 8,931,009 |
| **8** | TS=((perspective* OR experience* OR attitude* OR need* OR emotion* OR affect OR communication OR access* OR comprehension OR perception* OR opinion* OR view* OR satisfaction OR preference OR barrier* OR obstacle* OR facilitator* OR expectation*) NEAR/5 (healthcare OR "health care" OR health-care OR "medical care" OR hospital* OR "health service*" OR "medical service*" OR "primary care" OR inpatient* OR patient* OR "acute setting" OR "acute care" OR "care setting" OR ward* OR "emergency department*" OR "emergency service*" OR "health facilit*" OR "health care facilit*")) | 958,067 |
| **Combined sets** | | |
| **9** | #5 AND #8 | 1,813 |
| **Additional filters** | | |
| **18** | Document Types: Article; Publication Years (2014–) | 1,218 |

| Database: Medline via Ebsco  2024-03-25 | | Items found |
| --- | --- | --- |
| **Autistic adults** | | |
| **1** | MH Autism Spectrum Disorder OR MH Autistic Disorder | 43,612 |
| **2** | TI (autism* OR autistic* OR "autism spectrum disorder") OR AB (autism* OR autistic* OR "autism spectrum disorder") OR CI (autism* OR autistic* OR "autism spectrum disorder") | 73,802 |
| **3** | S1 OR S2 | 73,802 |
| **4** | MH Infant OR MH Child OR MH Adolescent OR MH Minors | 3,560,374 |
| **5** | TI (Infant* OR infancy OR Newborn* OR Baby* OR Babies OR Child* OR Schoolchild* OR School age* OR Kid OR kids OR Toddler* OR Adoles* OR Teen* OR Boy OR boys OR boyfriend OR boyhood OR Girl* OR Minors OR minor) OR AB (Infant* OR infancy OR Newborn* OR Baby* OR Babies OR Child* OR Schoolchild* OR School age* OR Kid OR kids OR Toddler* OR Adoles* OR Teen* OR Boy OR boys OR boyfriend OR boyhood OR Girl* OR Minors OR minor) OR CI (Infant* OR infancy OR Newborn* OR Baby* OR Babies OR Child* OR Schoolchild* OR School age* OR Kid OR kids OR Toddler* OR Adoles* OR Teen* OR Boy OR boys OR boyfriend OR boyhood OR Girl* OR Minors OR minor) | 5,053,934 |
| **6** | S4 OR S5 | 5,053,934 |
| **7** | MH Adult | 5,492,573 |
| **8** | TI (adult* OR “middle aged” OR elderly OR women OR men OR woman OR man) OR AB (adult* OR “middle aged” OR elderly OR women OR men OR woman OR man) OR CI (adult* OR “middle aged” OR elderly OR women OR men OR woman OR man) | 9,092,889 |
| **9** | S7 OR S8 | 9,092,889 |
| **10** | S6 NOT S9 | 2,712,180 |
| **11** | S3 NOT S10 | 37,674 |
| **Experience of health care** | | |
| **12** | MH “attitude to health” OR MH “health communication” OR MH “health knowledge, attitudes, practice” OR MH “patient preference” OR MH “patient satisfaction” OR MH “health services accessibility” | 380,747 |
| **13** | TI (perspective* OR experience* OR attitude* OR need* OR emotion* OR affect OR communication OR access* OR comprehension OR perception* OR opinion* OR view* OR satisfaction OR preference OR barrier* OR obstacle* OR facilitator* OR expectation*) OR AB (perspective* OR experience* OR attitude* OR need* OR emotion* OR affect OR communication OR access* OR comprehension OR perception* OR opinion* OR view* OR satisfaction OR preference OR barrier* OR obstacle* OR facilitator* OR expectation*) OR CI (perspective* OR experience* OR attitude* OR need* OR emotion* OR affect OR communication OR access* OR comprehension OR perception* OR opinion* OR view* OR satisfaction OR preference OR barrier* OR obstacle* OR facilitator* OR expectation*) | 7,576,701 |
| **14** | TI (healthcare OR “health care” OR health-care OR “medical care” OR hospital* OR “health service*” OR “medical service*” OR “primary care” OR inpatient* OR patient* OR “acute setting” OR “acute care” OR “care setting” OR ward* OR “emergency department*” OR “emergency service*” OR “health facilit*” OR “health care facilit*”) OR AB (healthcare OR “health care” OR health-care OR “medical care” OR hospital* OR “health service*” OR “medical service*” OR “primary care” OR inpatient* OR patient* OR “acute setting” OR “acute care” OR “care setting” OR ward* OR “emergency department*” OR “emergency service*” OR “health facilit*” OR “health care facilit*”) OR CI (healthcare OR “health care” OR health-care OR “medical care” OR hospital* OR “health service*” OR “medical service*” OR “primary care” OR inpatient* OR patient* OR “acute setting” OR “acute care” OR “care setting” OR ward* OR “emergency department*” OR “emergency service*” OR “health facilit*” OR “health care facilit*”) | 10,253,867 |
| **15** | S13 N5 S14 | 1,096,840 |
| **16** | S12 OR S15 | 1,260,877 |
| **Combined sets** | | |
| **17** | S11 AND S16 | 1,523 |
| **Additional filters** | | |
| **18** | Academic Journals; Peer Reviewed; Publication Date (2014–) | 1,083 |

| Database: Scopus 2024-03-25 | | Items found |
| --- | --- | --- |
| **Autistic adults** | | |
| **1** | TITLE-ABS-KEY (autism* OR autistic* OR "autism spectrum disorder") | 122,589 |
| **2** | TITLE-ABS-KEY (infant* OR infancy OR newborn* OR baby* OR babies OR child* OR schoolchild* OR school AND age* OR kid OR kids OR toddler* OR adoles* OR teen* OR boy OR boys OR boyfriend OR boyhood OR girl* OR minors OR minor) | 2,926,545 |
| **3** | TITLE-ABS-KEY (adult* OR "middle aged" OR elderly OR women OR men OR woman OR man) | 12,228,845 |
| **4** | S1 AND NOT S2 | 78,920 |
| **5** | S1 AND NOT S4 | 43,669 |
| **Experience of health care** | | |
| **6** | TITLE-ABS-KEY (perspective* OR experience* OR attitude* OR need* OR emotion* OR affect OR communication OR access* OR comprehension OR perception* OR opinion* OR view* OR satisfaction OR preference OR barrier* OR obstacle* OR facilitator* OR expectation*) | 20,682,323 |
| **7** | TITLE-ABS-KEY (healthcare OR "health care" OR health-care OR "medical care" OR hospital* OR "health service*" OR "medical service*" OR "primary care" OR inpatient* OR patient* OR "acute setting" OR "acute care" OR "care setting" OR ward* OR "emergency department*" OR "emergency service*" OR "health facilit*" OR "health care facilit*") | 13,521,257 |
| **8** | TITLE-ABS-KEY (perspective* OR experience* OR attitude* OR need* OR emotion* OR affect OR communication OR access* OR comprehension OR perception* OR opinion* OR view* OR satisfaction OR preference OR barrier* OR obstacle* OR facilitator* OR expectation* ) W/5 TITLE-ABS-KEY ( healthcare OR "health care" OR health-care OR "medical care" OR hospital* OR "health service*" OR "medical service*" OR "primary care" OR inpatient* OR patient* OR "acute setting" OR "acute care" OR "care setting" OR ward* OR "emergency department*" OR "emergency service*" OR "health facilit*" OR "health care facilit*") | 1,489,946 |
| **Combined sets** | | |
| **9** | S5 AND S8 | 2,054 |
| **Additional filters** | | |
| **18** | Document type: Article; Publication Year (2014–) | 1,268 |
